# Iodine Status of New Zealand Elderly Residents in Long-Term Residential Care

**DOI:** 10.3390/nu8080445

**Published:** 2016-07-23

**Authors:** Jody C. Miller, Sue O. MacDonell, Andrew R. Gray, Malcolm R. Reid, David J. Barr, Christine D. Thomson, Lisa A. Houghton

**Affiliations:** 1 Department of Human Nutrition, University of Otago, P.O. Box 56, Dunedin 9054, New Zealand; sue.macdonell@otago.ac.nz (S.O.M.); christine.thomson@otago.ac.nz (C.D.T.); lisa.houghton@otago.ac.nz (L.A.H.); 2 Department of Preventive and Social Medicine, University of Otago, P.O. Box 56, Dunedin 9054, New Zealand; andrew.gray@otago.ac.nz; 3 Trace Element Centre, Department of Chemistry, University of Otago, P.O. Box 56, Dunedin 9054, New Zealand; malcolm.reid@otago.ac.nz (M.R.R.); david.barr@otago.ac.nz (D.J.B.)

**Keywords:** iodine, urinary iodine, thyroglobulin, elderly

## Abstract

In response to the re-emergence of iodine deficiency in New Zealand, in 2009 the government mandated that all commercially made breads be fortified with iodized salt. There has been no evaluation of the impact of the program on iodine status of the elderly, despite this population group being vulnerable to iodine deficiency or excess. The aim of this study was to describe the iodine status of elderly New Zealanders in residential aged-care homes following the implementation of the bread fortification program. A cross-sectional survey was conducted, involving 309 residents (median age 85 years) from 16 aged-care homes throughout NZ. Information on socio-demographic, anthropometric, dietary and health characteristics were collected. Casual spot urine samples were analysed for urinary iodine concentration (UIC). Blood samples were analysed for serum thyroglobulin, thyroglobulin antibodies, and other biochemical indices. The median UIC (MUIC) of the residents was 72 μg/L, indicating mild iodine deficiency, and 29% had a UIC < 50 μg/L. Median thyroglobulin concentration was 18 ng/mL and 26% had elevated thyroglobulin concentration (>40 ng/mL), suggesting iodine insufficiency. Diuretic use was associated with lower MUIC (*p* = 0.043). Synthetic thyroxine use was associated with lower odds of having a UIC < 50 μg/L (OR 0.32, *p* = 0.030)) and lower median thyroglobulin (−15.2 ng/mL, *p* = 0.001), compared with untreated participants. Frailty was associated with elevated thyroglobulin (*p* = 0.029), whereas anemia was associated with lower thyroglobulin (*p* = 0.016). Iodine insufficiency persists in New Zealanders residing in residential aged-care homes despite increasing iodine intake from fortified bread. Research is required to establish optimal iodine intake and status in the elderly.

## 1. Introduction

Iodine is an essential component of thyroid hormones, which are required for maintenance of tissues and regulation of the metabolic rate. Inadequate iodine intake can lead to thyroid dysfunction, which in turn causes a range of adverse health conditions collectively referred to as Iodine Deficiency Disorder [1]. Although the detrimental health effects of iodine deficiency are most pronounced in the fetus and during infancy, adverse effects are observed at all stages of the life cycle [1,2]. Thyroid dysfunction in adults is associated with a number of important adverse health outcomes, including hypertension, dyslipidemia, cognitive impairment, osteoporosis, muscle wasting, frailty, and neuromuscular dysfunction [3]. Older adults are at increased susceptibility to iodine deficiency or excess due to age-related changes in thyroid function. For example, thyroid autonomous nodules are increased in older adults who reside in areas of mild to moderate iodine deficiency, and are associated with subclinical hyperthyroidism when iodine intake is low, or after increases in intake following implementation of iodine fortification programs [2].

The elderly are a rapidly growing segment of the population, and it is, therefore, a high public health priority to ensure older adults have sufficient iodine intake to maintain optimal thyroid function and reduce the burden on health care resources. In New Zealand, prior to the 1940s, goiter was endemic in many parts of the country, but was almost eliminated after a salt iodization program was implemented in 1939, whereby table salt was iodized at 50 ppm [4]. However, iodine deficiency re-emerged in New Zealand in the late 1990s [5,6], resulting from changes in dietary habits, including declines in discretionary use of iodized salt, and increased intake of processed foods that usually do not contain iodized salt [7]. Consequently, the New Zealand government mandated that all commercially made breads be fortified with iodized salt from September 2009, with the aim to increase iodine intakes [4]. Since the implementation of the bread fortification program, a modest improvement in the iodine status of children and adults has been reported [8,9]; however, no studies have assessed the impact of the fortification program on iodine intake or status of elderly New Zealanders. Older adults receiving long-term residential care are at particularly high risk of nutrient deficiencies because they have small appetites and are often unable to consume enough food to meet their nutrient requirements [10,11]. Furthermore, they often have both acute and chronic diseases, and use medications or oral nutritional supplements that may increase the risk of suboptimal or excessive nutrient status due to varying nutrient intake, absorption, metabolism and/or excretion [12,13]. For example, older institutionalized adults with renal insufficiency may have lower iodine requirements from decreased excretion of iodine. Therefore, while previous studies have focused on monitoring the effect of the mandatory bread fortification program on the iodine intake or status of younger populations [9,14], it is also important to evaluate the impact of the program on the elderly.

In 2014, we conducted a cross-sectional survey of older adults in residential-care homes across New Zealand, in order to investigate the nutritional status and health of these residents. As part of a full nutritional assessment, dietary iodine intake and biochemical iodine status were measured. Thus, the aim of the present study was to describe the iodine status of elderly New Zealanders in long-term residential care following the implementation of the mandatory fortification of bread program, and to estimate the impact of the program on dietary iodine intakes.

## 2. Materials and Methods

The New Zealand Nutrition and Ageing Project was a cross-sectional survey, involving 309 long-term residents from residential-care homes (rest homes) throughout New Zealand. Twenty rest homes were identified from two national aged-care websites, based on having sufficient numbers of residents to recruit approximately 20 participants in each home. Managers of 16 homes agreed to participate, from Auckland, Hamilton, Hawkes Bay, Wellington, Christchurch, Dunedin and Invercargill. Residents who had resided in the home for ≥12 weeks, were aged 60 years and older (with no upper age limit applied), and were receiving residential-level care, were eligible to participate. In New Zealand, rest home level care is for adults who require 24 h supervision with activities of daily living but are generally able to self-feed and are mobile with supervision. Participants were recruited with assistance from rest home staff and by posting posters and information sheets around the rest home. Interested participants were given an information sheet detailing the study protocol. Written, informed consent was obtained from all participants or from a legal representative for those with cognitive impairment. Ethical approval for the study was obtained from the University of Otago Human Ethics (Health) Committee (H13/118).

Trained research assistants undertook data collection during two time points in 2014. The first phase was conducted between February to April (late summer/autumn), and the second phase between July to September (late winter/spring). Demographic data (age, sex, ethnicity, duration of residency in the home) and smoking status (current smoker or non-smoker) were collected from medical records and participants using pre-tested questionnaires. Medical history, prescribed regular medications (medications prescribed pro re nata were not recorded), oral nutritional supplements, and vitamin/minerals supplements use (type, frequency and dose) were recorded from medical records held at the rest home. Participants who were prescribed levothyroxine, antithyroid medications (carbimazole), had a physician diagnosis of recent hyper-, or hypothyroidism, or had ever had a thyroidectomy were classified as having thyroid dysfunction.

Body weight and ulna length (a surrogate estimate of height [15]) were taken according to standardized procedures. Height estimated from ulna length was used for this study, because many participants were unable to stand under a stadiometer to collect accurate standing height measures. Ulna length was measured to the nearest 0.1 cm, and converted to height using published conversion charts [15]. Weight was measured to the nearest 100 g using Seca digital scales (range 2–200 kg) placed on a hard surface, with participants wearing light clothing and without footwear. For participants unable to stand on the scales, rest home seated scales that were calibrated against the standard study scales were used. Body mass index (BMI) was calculated from estimated height and measured weight (kg/m^2^).

Malnutrition risk was determined using the Malnutrition Universal Screening Tool (MUST) [15] with information collected from medical and rest home records. This tool uses current BMI, weight loss over the previous 3–6 months and acute illness with no nutritional intake over the previous five days to calculate a risk score. A score of 0 indicates a low risk of malnutrition, a score of 1 indicates medium risk and scores of 2–6 indicate high risk.

Frailty scores were determined using the Survey of Health, Ageing and Retirement in Europe Frailty Instrument (SHARE-FI) [16]. Participants were assigned a score based on five variables: (1) self-reported exhaustion; (2) self-reported diminution in desire for food; (3) weakness, assessed by measured handgrip strength; (4) self-reported slowness; and (5) self-reported low activity. Total scores were subsequently categorized as non-frail, pre-frail or frail according to the sex-specific cut-offs validated by Romero-Ortuno and colleagues [16]. Handgrip strength was measured using JAMAR PLUS+ digital dynamometers (Model J000105 Hydraulic Hand Dynamometer, Rolyon, IL, USA), with readings taken three times from each hand and the highest score from any hand used in calculating frailty risk.

Dietary intake data were collected by trained research assistants via weighed, three-day diet records (3DDR) using Salter electronic scales accurate to within ±1 g (Salter Housewares Ltd., Tonbridge, UK, range 2–2000 g). Diet recording was conducted on non-consecutive days, and covered both week (two) and weekend (one) days. During the 3DDR, all food and beverage items served to the participant by the rest home food service for breakfast, lunch, dinner and morning and afternoon tea were weighed and recorded before each meal, and any leftovers were weighed to determine actual amount consumed. The 3DDR included any energy containing oral nutritional supplements consumed (e.g., Ensure, Complan). Additionally, participants were asked prior to mealtimes and the morning following the diet-recording day to recall any foods or beverages consumed during the 24 h or throughout the evening that may not have been weighed and recorded. Portion sizes for this recall were based on the participant’s usual serving size when in the rest home. Rest home staff recorded foods consumed by the participant during supper (an evening snack served at around 7.00 p.m.). Detailed recipes of menu items served to participants were collected from the rest home’s food service. Participants were instructed to use food diaries to record all food and beverages consumed when away from the rest home. A research assistant then discussed and checked these with the participant on their return to the rest home and verified records were included in the 3DDR.

Food composition data are not complete for iodine, and therefore measurement of dietary intake from diet records is not accurate. Instead, median urinary iodine concentration (MUIC) is a better estimate of iodine intake in a population. However, it was possible to estimate the amount of iodine in bread pre-fortification, and the additional intake post-fortification. Individual diet records were examined to identify the amount and types of breads consumed daily. The amount of iodine obtained from commercially made bread was then calculated (μg iodine/gram bread) using analytically determined values for the specific type of bread (e.g., white, wholemeal, rye) reported in the Total Diet Survey for pre-fortification values [17], and by the Ministry of Agriculture and Forestry for post-fortification values [4].

### 2.1. Biochemical Analyses

Iodine status was assessed using urinary iodine concentration determined from casual, spot urine samples, and serum thyroglobulin concentration. Because 90% of ingested iodine is excreted in urine, urinary iodine concentration is a good biomarker of iodine intake. However, there are large day-to-day variations in iodine intake, and repeated 24 h urine samples are required to accurately assess an individual’s usual iodine intake and status. It was not logistically possible to collect 24 h urine samples in this study; therefore, we used the MUIC from spot urine samples to estimate population iodine status. This is recognized as a valid indicator of population iodine status if the sample size is sufficient (100 to 500 participants) [18] to off-set inter-individual variations in hydration and urine volume, and intra-individual day-to-day variations in iodine intakes. Serum thyroglobulin concentration is a promising functional biomarker of iodine status, and elevated levels indicate increased thyroid volume and thyroid hyperplasia associated with insufficient or excess iodine intake [19]. However, thyroglobulin antibodies (TgAb), which are commonly elevated in older individuals, interfere with thyroglobulin assays. We therefore also measured TgAb, to control for this interference.

Casual spot urine samples were collected in sterile plastic specimen containers from 250 participants, and transported on ice to the Department of Human Nutrition, University of Otago, Dunedin, where they were stored at −20 °C until analysis. Urine samples were not collected from participants in two rest homes (*n* = 40 participants) due to logistical constraints, and a further 19 participants did not provide urine samples due to difficulties in voiding or refusal to provide a sample. Urinary iodine concentration was measured on an Agilent 7500ce ICP-MS (Agilent Technologies, Santa Clara, CA, USA) in the Trace Element Centre, Department of Chemistry, University of Otago. This laboratory participates in Ensuring the Quality of Urinary Iodine Procedures (EQUIP) quality assurance program conducted by the United States Centers for Disease Control and Prevention. Urine samples were diluted 1:20 with 1% (*v*/*v*) tetra-methylammonium hydroxide, and 0.01% Triton X-100™ solution spiked with tellurium as a reference element. Calibration solutions were prepared by spiking a pooled sample with various concentrations of analyte prepared from a NIST traceable 10 mg/mL iodine standard. Samples with elevated UIC (>800 µg/L) were diluted with >18.2 MΩcm water and rerun. Analyses of a commercial control (Seronorm Trace Elements Urine, Lot no. 1011645, Sero AS, Asker, Norway) with an assigned iodine concentration of 304 µg/L and an acceptable range of 260–348 µg/L, gave a mean (SD) of 284 (4) µg/L (*n* = 13, coefficient of variation (CV) 1.3%). Regular measurements of an in-house pooled urine control gave a mean (SD) of 57 (1) µg/L (*n* = 33, CV 2.4%).

Fasting venous blood samples were drawn into an additive free vacutainer for determining serum thyroglobulin, TgAb, and high-sensitivity C-Reactive Protein, and serum creatinine for determining estimated Glomerular Filtration Rate (eGFR). In addition, blood was drawn into an EDTA-treated vacutainer for a complete blood count (including hemoglobin), which was undertaken immediately by a local health clinic. The remaining samples were transported chilled to the University of Otago, on the same day of collection, where they were immediately centrifuged for 15 m at 4 °C. Aliquots of serum were frozen at −80 °C until analysis.

Serum thyroglobulin and TgAb concentrations were determined using an electrochemiluminescent immunoassay (Roche) on an Elecsys 2010 autoanalyser (Tokyo, Japan). The standards for the Roche thyroglobulin assay have been standardized against the international reference material CRM-457. The analytical range for thyroglobulin was 0.04 to 500 ng/mL, and 10 to 4000 IU/mL for TgAb. Analysis of controls supplied by the manufacturer gave a mean (SD) of 18.4 (0.8) ng/mL for the normal thyroglobulin control (expected range 13.9 to 24.1 ng/mL), and 75.3 (1.2) ng/mL for the high thyroglobulin control (expected range 54.1 to 94.1 ng/mL). The mean (SD) for the normal TgAb control was 101.9 (3.8) IU/mL (expected range 69.0 to 128.2 IU/mL), and 219.8 (5.6) IU/mL for the high TgAb control (expected range 158.3 to 293.8 IU/mL). Analysis of a pooled serum sample within each batch gave a mean (SD) of 28.0 (0.5) ng/mL (CV 1.7%) and 30.5 (2.9) U/mL (CV 9%) for thyroglobulin and TgAb, respectively.

High-sensitivity C-Reactive Protein (hs-CRP), a measure of acute inflammation, was determined by automated immunoturbidimetric assay (Cobas c311; Roche Diagnostics). Controls provided by the manufacturer (PCCM1) were analyzed with each reagent kit. The mean (SD) for the control was 10.10 (0.14) g/L (CV 1.40), and were within the range of the results provided by the manufacturer.

Serum creatinine was measured using Creatinine Jaffé Gen.2 kinetic colorimetric assay (Cobas c311; Roche Diagnostics). Controls provided by the manufacturer (PreciControl ClinChem Multi 1 and PreciControl ClinChem Multi 2) had target values of 97.8 µmol/L and 365 µmol/L respectively and were analyzed daily. The means and CV (%) for the two controls were 96 (SD 1.0) µmol/L and 1.04% and 367.78 (SD 5.76) µmol/L and 1.57% respectively. Estimated Glomerular Filtration Rate (eGFR), an indicator of renal function, was determined for each participant using serum creatinine, age and sex. These variables were entered in the online Chronic Kidney Disease Epidemiology collaboration (CKD-EPI) eGFR calculator [20] to obtain the eGFR for each participant. Categories of eGFR were then assigned using the Kidney Disease: Improving Global Outcomes (KDOGI) classifications as an indicator of renal function [21]. That is, lower eGFR indicates impaired renal function.

### 2.2. Statistical Analysis

Statistical analyses were conducted using Stata (Statacorp, LP, version 12). Statistical significance was determined by two-sided *p* < 0.05. All analyses included rest home sites as clusters using Froot’s extension to Huber-White robust standard errors [22,23].

The distributions of urinary iodine and serum thyroglobulin concentrations were positively skewed, and therefore medians (25th and 75th percentile) are reported. Based on the WHO/UNICEF/ICCIDD criteria, population MUIC within the range of 20–49 μg/L is indicative of moderate iodine deficiency, 50–99 μg/L as mild deficiency, and 100–199 μg/L as adequate iodine nutrition, 200–299 μg/L as above requirements, and >300 μg/L as excessive [24]. Iodine deficiency based on serum thyroglobulin concentration was defined as a median concentration >13 ng/mL and/or more than 4% with serum thyroglobulin concentration >40 ng/mL [25,26], after excluding participants with serum TgAb concentration >115 IU/mL. The cut-off for TgAb was based on the reference range established by the manufacturer for healthy euthyroid individuals.

Unadjusted quantile regression models were used to initially examine the effect of sex, age, time of data collection, BMI, malnutrition risk, frailty, medications (specifically loop diuretics and levothyroxine), oral supplement use, and anemia, on urinary iodine and serum thyroglobulin concentrations. Unadjusted logistic regression models were then used to examine the effect of these variables on the odds of having low urinary iodine concentration (<50 μg/L) or elevated serum thyroglobulin concentration (>40 ng/mL). The explanatory variables included in the models were those known or suspected to influence nutrient intake [27,28] or iodine status [25,29,30,31]. Anemia was defined using the World Health Organization cut-offs for hemoglobin of <120 g/L and <130 g/L for women and men, respectively [32]. Quantile and logistic regression models containing all independent variables with a *p*-value < 0.25 from the unadjusted analysis were constructed. The number of variables included in the adjusted logistic regression models was constrained by the 10 EPV rule [33]. The only plausible interaction examined was that of sex-by-BMI, which was added to each adjusted model and retained if statistically significant.

Sensitivity analyses were performed excluding smokers (*n* = 9 with both urinary iodine and Tg results, this was tested because smoking may increase serum thyroglobulin concentration), those on amiodarone (*n* = 2, this was tested due to the high urinary iodine levels), and those prescribed levothyroxine or diagnosed with a thyroid disorder (*n* = 35).

## 3. Results

Selected characteristics for all participants, and for those with and without a urinary iodine and serum thyroglobulin result, are presented in Table 1. The median age was 85 years, ranging from 65 to 107 years, with 68% women. Almost all participants (98%) were of New Zealand European ethnicity, and 3 identified as Māori. The average duration of residence in the rest home was 31 months, ranging from 2 months to 13 years.

Of the 60 participants diagnosed with any thyroid disorder, 9 were diagnosed with hyperthyroidism, 46 with hypothyroidism, and 5 had a partial thyroidectomy. Fifty participants (16%) were prescribed levothyroxine (synthetic thyroxine) and four prescribed anti-thyroid medications. On average, participants were prescribed a total of 6 regular medications (range 0 to 15). Of the 112 participants prescribed loop diuretics, most (98%, *n* = 110) were prescribed furosemide. Two participants were prescribed amiodarone (antiarrhythmic agent with beta blocker-like actions), providing 75 mg of iodine per day. Oral nutritional supplements were prescribed for 22 (7%) of participants, contributing an average 14 µg of iodine per day, and 29 (9%) were taking supplements containing iodine (e.g., multivitamin supplements). Participants at high risk of malnutrition were more likely to be prescribed oral nutritional supplements (24% for high risk, 4% for moderate risk, and 1% for low risk, *p* = 0.007). The majority of participants (83%) were identified as being either pre-frail or frail. There were no meaningful differences in characteristics between participants with and without a urinary iodine and serum thyroglobulin result.

The MUIC of participants was 72 μg/L, and 29% had a urinary iodine concentration <50 μg/L at the time of the study, indicating mild iodine deficiency in this population based on the WHO/UNICEF/ICCIDD criteria [24] (Table 2). Eleven participants (4%) had a urinary iodine concentration >300 μg/L (considered “excessive”), two of whom were prescribed amiodarone and had extremely high urinary iodine levels (3761 and 4965 µg/L). Excluding these two participants from the analysis, however, did not meaningfully alter the results reported herein. Urinary iodine concentrations were lower in summer/autumn, in the oldest age group, and in participants with stage 3 and 4 chronic kidney disease. Participants treated with levothyroxine had significantly higher MUIC (*p* = 0.028), lower percentage with a concentration <50 μg/L (*p* = 0.031), and a higher proportion with urinary iodine concentration >300 μg/L (*p* = 0.017), compared with participants not on this medication.

After excluding participants with elevated TgAb (*n* = 35, 13%), the median thyroglobulin concentration was 18 ng/mL and 26% had an elevated thyroglobulin concentration (>40 ng/mL). Both of these values were above the assay-specific recommendations to indicate population iodine sufficiency (median serum thyroglobulin <13 ng/mL, and/or no more than 4% with thyroglobulin concentration >40 ng/mL [26]) further indicating that the iodine status of this population was insufficient. The prevalence of elevated thyroglobulin concentrations was highest in women and pre-frail participants. In participants treated with levothyroxine, the median serum thyroglobulin concentration of 7 ng/mL was within recent guidelines, but the proportion with elevated thyroglobulin (16%) was higher than recommended [26].

In the adjusted analyses (Table 3), median MUIC was higher during summer/autumn (*p* = 0.004), with a non-significant tendency for lower odds of having a urinary iodine concentration <50 µg/L (*p* = 0.056), than during winter/spring. Participants on loop diuretics had lower median MUIC (*p* = 0.043), and those on levothyroxine had lower odds of having a low urinary iodine concentration (*p* = 0.030), compared with untreated participants. For serum thyroglobulin (Table 4), adjusted analysis showed median thyroglobulin concentration increased with age and with BMI, and there was a non-significant tendency for a positive association with hs-CRP (*p* = 0.057). Frailty was associated with higher odds of having elevated serum thyroglobulin concentration, whereas anemic patients and those treated with levothyroxine had lower odds.

In a sensitivity analysis, MUIC was slightly lower (65 µg/L) when participants who were prescribed levothyroxine and thyroid disorders were excluded (*n* = 35). However, the results for serum thyroglobulin remained largely unchanged. Excluding participants with thyroid disorders or on levothyroxine, or excluding smokers or those on amiodarone, did not appreciably alter the results from the adjusted analyses (data not shown). The mean intake of bread per day was 66 g/day, equivalent to approximately 2 slices of bread (~30 g per slice) (Table 5). Bread intake was significantly higher in men compared with women (*p* = 0.001). Bread intake was inversely associated with age, malnutrition risk and frailty, and was positively associated with BMI (*p* = 0.003). Fortification of bread with iodized salt increased the mean intake of iodine from bread by 31 μg/day.

## 4. Discussion

The findings from our nation-wide survey indicate that despite the implementation of mandatory fortification of commercially baked bread with iodized salt in late 2009, iodine insufficiency persists in this elderly population group residing in long-term residential care. Participants were classified as mildly iodine deficient, based on internationally recognized criteria recommend by the WHO/UNICEF/ICCIDD for assessing population iodine adequacy with urinary iodine concentration (MUIC between 100 and 199 μg/L, with no more than 20% of samples below 50 μg/L [24]). Further indication of iodine insufficiency was evident from elevated serum thyroglobulin concentrations, with both the median serum thyroglobulin concentration (18 ng/mL) and percentage with elevated levels (26%) exceeding recent recommendations to indicate population iodine sufficiency [median thyroglobulin <13 ng/mL, and/or <4% of the population with thyroglobulin concentration >40 ng/mL] [26].

While our finding of iodine insufficiency in this elderly population is strengthened by the use of two objective biochemical indices of iodine status, our results should be interpreted with caution because each index individually has limitations that hinder interpretation in older adults. Specifically, although MUIC is the most widely used index for determining iodine status of a population [24], the criteria for defining iodine sufficiency with MUIC have been investigated predominantly in school-aged children and have not been adequately validated in adults [34]. The cut-off for MUIC recommended by WHO/UNICEF/ICCIDD for defining iodine sufficiency was based on a study in Central America, showing goiter rates to be <10% in areas where mean estimated 24 h urinary iodine excretion (UIE) was >100 μg [34]. The UIE cut-off was extrapolated to the MUIC, and when urine volume is approximately 1 L/day, as in school aged children, mean UIE and MUIC are interchangeable. However, higher urine volumes in adulthood (average 2 L for New Zealand adults [8]) would dilute urinary iodine concentrations. Thus, some researchers suggest that a MUIC of 60–70 μg/L would better correspond with a mean UIE of 100 μg to indicate iodine sufficiency for adults [34]. If this lower cut-off were also appropriate for older institutionalized adults, the iodine status of the participants in the present study would be deemed sufficient. Nonetheless, we also observed elevated serum thyroglobulin concentrations, which indicate thyroid hyperactivity potentially due to iodine insufficiency. Similar to urinary iodine concentration, however, there is also insufficient information for determining valid serum thyroglobulin reference ranges for adults [19]. Serum thyroglobulin concentrations increase with age, smoking, alcohol intake, thyroid disorders, and the presence of thyroid nodules, in addition to iodine insufficiency [25]. A limitation of our study is that we did not examine thyroid function or presence of thyroid nodules. Few participants in our study were current smokers or drank alcohol, and controlling for age or excluding smokers or those with diagnosed thyroid disorders did not alter our findings. The close agreement between the percentage with low urinary iodine concentration (29% <50 μg/L) and elevated serum thyroglobulin (26%) tends to support a lower cut-off for older adults, and that iodine insufficiency is prevalent in this population. Nevertheless, randomized controlled trials are needed to elucidate the relation between MUIC cut-offs and serum thyroglobulin in adults, and their relation with thyroid hormone disorders.

Further exacerbating the difficulties in assessing iodine status in institutionalized adults is the high prevalence of disease states or use of medications that may alter nutrient metabolism and nutrient requirements, and influence the validity of nutrient biomarkers. Renal insufficiency decreases urinary iodine excretion through reduced renal filtration of iodine from plasma and increased tubular resorption of the filtered iodine, leading to high levels of iodine in plasma [35]. Therefore, in patients with renal insufficiency low urinary iodine concentration does not necessarily indicate poor iodine status. In the present study, while we observed lower median urinary iodine concentrations in participants with chronic kidney disease, serum thyroglobulin concentrations were not statistically associated with renal function. It is therefore possible that higher supply of plasma iodine with impaired renal excretion maintained thyroid function. Nevertheless, our ability to detect differences in iodine status between stages of chronic kidney disease may be limited by the potential for misclassification of renal function in some participants since we used serum creatinine and established equations to calculate eGFR. Serum creatinine levels are affected by age, lean body mass and undernutrition, particularly low protein intakes [36]. Although not measured in our study, many participants are likely to have sarcopenia (low lean body mass), and many had very low protein intakes (data not shown), thereby limiting the accuracy of eGFR calculations that are based on healthy, well-nourished individuals.

In the current study, loop diuretic use such as furosemide (most commonly prescribed) was associated with lower MUIC, but not with serum thyroglobulin concentration. It is possible that increased urinary volume with diuretic use may have diluted urinary iodine concentration. Furthermore, use of furosemide in combination with a low iodine diet may increase thyroidal uptake of iodine [37], thus reducing iodine excretion and maintaining normal thyroid function. Conversely, treatment with levothyroxine (synthetic thyroxine hormone) was associated with higher MUIC, including higher prevalence of excessive urinary iodine intake (urinary iodine concentration >300 μg/L), and lower median thyroglobulin concentration. It is likely that the dietary iodine requirements of patients on levothyroxine are lower, because removal of the need for endogenous production of thyroxine would lower physiological iodine requirements. Moreover, additional iodine would be made available during the deiodination of synthetic thyroxine to the biologically active thyroid hormone, triiodothyronine. While other studies have excluded participants on synthetic thyroid hormone treatment when assessing iodine status of adults, we elected to include these participants comprising 13% of study population because our aim was to provide a representative description of the iodine status of this group. In institutionalized older adults, dietary iodine might not be the only, or main, source of iodine, and excluding the high percentage on levothyroxine might therefore slightly underestimate iodine status of our population, if status is indeed better when taking this medication.

Notwithstanding the above-mentioned limitations in assessing iodine status of the elderly, our findings provide important information on predictors of low iodine status in institutionalized elderly. That frail participants were at higher risk of elevated thyroglobulin concentration is of particular interest. Frailty is an important geriatric syndrome that substantially increases morbidity, health care costs, and reduces quality of life [38]. The criteria for frailty (weakness, exhaustion, slowness, poor exercise tolerance and/or unintentional weight loss [39]) overlap with the symptoms of thyroid dysfunction (fatigue, reduced muscle strength, and weight change [3]). Recent cross-sectional studies have found positive associations between high-normal thyroxine hormone concentration and frailty [40], or reduced physical function [41], in older men. Alternatively, TgAb positivity−which is a risk factor for hypothyroidism−was associated with lower risk of frailty in older women [42]. Whether the association between frailty and thyroid function is influenced by iodine intake is uncertain because iodine status was not measured in these earlier studies. Nutrients other than iodine might influence thyroid hormone synthesis (e.g., selenium [31]), which are often low in the diets of older adults [28,43]. Moreover, we did not measure thyroid hormones in the present study, nor did we detect any differences in MUIC between frail and non-frail participants, suggesting iodine intake did not differ by frailty status despite non-frail participants having higher average bread intake and estimated post-fortification iodine intake from bread. It is thus possible the relation between elevated serum thyroglobulin and frailty observed in our study is due to causes other than iodine deficiency. Nevertheless, if frailty is indeed influenced by thyroid hormone disorders, which may also be influenced by iodine intake, it is important to consider the impact of increasing iodine intake through fortification programs on thyroid health in older adults. In populations from historically iodine-deficient regions, transient increases in hyperthyroidism and use of antithyroid medications have been observed after the implementation of salt iodization programs [2,44]. This may be due to autonomously functioning thyroid tissue that develops in some individuals during periods of iodine deficiency [2]. While usually transient and asymptomatic in healthy adults, some deaths from iodine-induced hyperthyroidism after implementation of universal salt iodization in iodine deficient populations have been reported [41]. The clinical course of iodine-induced hyperthyroidism in frail elderly who have severely reduced resilience to physiological stressors is unknown. Further research is required to elucidate the associations between iodine intake, thyroglobulin and thyroid hormones on frailty risk in the elderly.

The lower risk of elevated serum thyroglobulin in anemic participants in our study was unexpected. Iron deficiency with or without anemia impairs the response of iodine deficient children to iodine prophylaxis [31]; therefore, if similar in adults, it would be expected that anemic older adults would have lower iodine status from impaired response to increasing iodine intake. Nevertheless, anemia in the elderly is mainly attributed to inflammation associated with chronic disease or of unexplained pathology rather than iron deficiency, per se [45]. We did not detect any effect of systemic inflammatory biomarkers (e.g., hs-CRP, interleukin 6, and alpha-1-acid glycoprotein (data not shown)) that are often associated with lower serum proteins, including hemoglobin, on the relation between serum thyroglobulin concentration and anemia. Our cross-sectional data, however, precludes determining causality. It would be of interest to further investigate the relation between anemia and serum thyroglobulin, given anemia increases with age, and might be an important confounder when assessing iodine status using thyroglobulin concentration in older adults.

While there are no previous data on iodine status of institutionalized New Zealand elderly, comparison of studies conducted pre- and post-fortification in other sectors of the population suggest iodine status has improved since mandatory fortification. The MUIC in the present study (72 μg/L) was slightly higher than the pre-fortification MUIC of 61 μg/L reported for community-dwelling older participants in the nationally representative 2008/2009 Adult Nutrition Survey [46] but the same as that reported for younger adults aged 18 to 64 years residing in Wellington and Dunedin in 2012, post-fortification [8]. In children, MUIC had increased from 68 μg/L pre-fortification in 2002 to 113 μg/L in 2010/2011, indicating post-fortification iodine sufficiency [9]. The replacement of salt in commercially baked breads with salt iodized at 25 to 65 μg iodine/kg of salt was estimated to provide an additional 78 μg of iodine per day for older adults aged 70 years and older [47]. We estimated an increase in iodine intake of approximately 31 μg/day from bread reflecting an average consumption of 2 slices per day. This is comparable to the estimated post-fortification increase of iodine intake from bread, of 35 to 37 μg/day in women of childbearing age [14] and in adults aged 18 to 64 years [8], but lower than the modeled estimate of 52 μg/day in children [4].

When developing the mandatory bread fortification policy in New Zealand, strategies such as mandating universal salt iodization, or replacing all salt with iodized salt in processed foods, or in breakfast cereals, biscuits and crackers in addition to bread, were considered [47]. These options were rejected after public consultation, because of perceived logistical difficulties in incorporating iodized salt into some food items, and risk of excessively high intakes for children in the case of universal salt iodization. However, extending mandatory use of iodized salt in other food items would be a good strategy to improve iodine intakes of older adults because they often have small appetites, and additional fortification would increase iodine intake without the need for an increase in food intake. This strategy should also improve iodine intakes of women of childbearing age, including those who are pregnant or lactating and are particularly vulnerable to inadequate intakes [14,48] although it would require careful surveillance of the iodine status of children to ensure excessive intakes are avoided. Another possible solution to improve iodine intakes in rest home residents is low dose supplementation. For example, in the present study the use of energy-containing oral nutritional supplements (e.g., Complan, Ensure) that provided an average of 14 μg of iodine per day was associated with lower odds of elevated thyroglobulin. Supplementation, however, is problematic because taste fatigue limits adherence, and polypharmacy increases the risk of nutrient-drug interactions. With an increased risk of hyperthyroidism with even a modest increase in iodine intake in the elderly with a history of iodine deficiency, any intervention should include close monitoring of adverse health effects. Worldwide, assessment of nutritional status in this lifecycle group is limited. The importance of such is particularly relevant in the context of global population ageing, with numbers of older adults escalating in regions with a history of iodine deficiency, including New Zealand, the UK, and some regions in Europe, Africa and Asia.

## Figures and Tables

**Table 1:** Characteristics of study participants.

	All Study Participants *n* (%)	With Urine and Blood Sample *n* (%)	Without Urine and Blood Sample *n* (%)
All participants	309	238	71
Women	209 (68)	162 (68)	47 (66)
Age, years			
<80	69 (22)	55 (23)	14 (20)
80–89	149 (48)	119 (50)	30 (42)
≥90	91 (30)	64 (27)	27 (38)
Time of data collection			
Summer/autumn	155 (50)	123 (52)	32 (45)
Winter/spring	154 (50)	115 (48)	39 (55)
Body Mass Index, kg/m^2^			
<20	42 (14)	29 (12)	13 (20)
20–24.9	104 (35)	82 (35)	22 (33)
25–29.9	95 (32)	74 (32)	21 (32)
≥30	60 (20)	50 (21)	10 (15)
Current smoker	12 (4)	9 (4)	3 (4)
Malnutrition risk ^a^			
Low risk	205 (70)	164 (72)	41 (62)
Moderate risk	53 (18)	38 (17)	15 (23)
High risk	37 (13)	27 (12)	10 (15)
Frailty ^b^			
Not frail	49 (17)	36 (16)	13 (21)
Pre-frail	93 (33)	74 (33)	19 (31)
Frail	143 (50)	113 (51)	30 (48)
Medications			
Loop diuretic	112 (36)	91 (38)	21 (30)
Laxative (regular prescription)	101 (33)	83 (35)	18 (25)
Levothyroxine	50 (16)	41 (17)	9 (13)
Beta-blocker	124 (40)	95 (40)	29 (41)
Oral nutritional supplement	22 (7)	16 (7)	6 (9)

^a^ Malnutrition risk assessed using the Malnutrition Universal Screening Tool [15]; ^b^ Frailty scores were determined using the Survey of Health, Ageing and Retirement in Europe Frailty Instrument (SHARE-FI) [16].

**Table 2:** Urinary iodine and serum thyroglobulin concentration of rest home residents in New Zealand.

Urinary Iodine (µg/L)							TgAb > 115 IU/mL *%*	Thyroglobulin (ng/mL) *			
	*n*	Median	25th, 75th Percentile	<50%	<100%	>300%		*n*	Median	25th, 75th Percentile	>40%
All	249	72	43, 109	29	71	4	13	232	18	9, 43	26
Sex											
Men	79	77	45, 112	27	70	5	8	77	16	9, 26	18 ^a^
Women	170	71	43, 105	30	71	4	15	155	21	9, 49	30 ^b^
Age group (years)											
<79	56	81 ^a^	51, 149	23	61	5	12	52	15	9, 29	17
80–89	125	71	42, 108	30	70	6	11	119	19	9, 39	24
≥90	68	71 ^b^	44, 93	31	79	1	19	61	19	9, 75	36
Time of data collection											
Summer/autumn	128	63 ^a^	40, 98	38 ^a^	76	3	12	119	20	9, 43	27
Winter/spring	121	84 ^b^	53, 137	20 ^b^	65	6	14	113	16	9, 38	25
Body Mass Index (kg/m^2^)											
<20	30	69	44, 111	27	73	0	11	32	15	7, 32	22
20–24.9	87	70	42, 104	30	74	3	15	73	16	9, 31	22
25–29.9	76	77	47, 115	28	70	4	15	73	21	9, 45	30
≥30	53	77	40, 125	30	64	9	9	48	18	12, 45	27
Malnutrition risk											
Low risk	172	72	44, 112	28	69 ^a^	4	14	155	18	9, 31	28
Moderate risk	40	73	38, 93	35	83 ^b^	5	5	41	19	9, 45	20
High risk	28	70	44, 107	29	75	4	23	24	18	12, 45	21
Frailty											
Non-frail	40	66	42, 95	33	75	5	13	42	18	10, 30	14 ^a^
Pre-frail	77	81	41, 117	30	68	5	10	88	18	8, 49	36 ^b^
Frail	117	70	43, 105	30	73	4	13	136	18	7, 33	24
Chronic kidney disease											
No disease	11	85 ^a^	70, 111	9 ^a^	73	0	14	12	11 ^a^	8, 18	8
Stage 1	115	73 ^a^	43, 129	30	66	5	14	109	18 ^b^	9, 43	26
Stage 2	106	72 ^a^	43, 100	27	75	4	13	95	19	8, 45	27
Stage 3/4	10	45 ^b^	32, 89	60 ^b^	80	10	7	14	23	9, 54	36
Taking Levothyroxine medication											
No	207	69 ^a^	42, 105	32 ^a^	74 ^a^	3 ^a^	11 ^a^	201	19 ^a^	8, 45	27
Yes	42	95 ^b^	59, 153	14 ^b^	55 ^b^	12 ^b^	26 ^b^	31	7 ^b^	2, 29	16
Taking oral nutritional supplement											
No	233	71	43, 107	30	72	5	13	217	19	9, 45	27
Yes	16	88	57, 176	19	56	0	21	15	12	9, 21	7

Abbreviations: TgAb, thyroglobulin antibody. * Participants with TgAb > 115 IU/mL (*n* = 35) were excluded from estimates. ^a,b^ Mean values or percentages within a subgroup column with unlike superscript letters were significantly different (*p* < 0.05).

**Table 3:** Predictors of urinary iodine concentrations of New Zealand rest home residents *.

	Urinary Iodine Concentration (μg/L)						Urinary Iodine Concentration <50 μg/L					
	Unadjusted			Adjusted ^a^			Unadjusted			Adjusted ^a^		
	Coef ^b^	95% CI	*p*-Value	Coef ^b^	95% CI	*p*-Value	OR	95% CI	*p*-Value	OR	95% CI	*p*-Value
Age (5-year increase)	−0.75	−4.40, 2.90	0.688				1.08	0.93, 1.26	0.331			
Sex (women cf men)	−6.02	−24.99, 12.96	0.533				1.18	0.56, 2.52	0.661			
Date of data collection (autumn/winter)	20.77	5.66, 35.88	0.007	23.24	7.29, 39.18	0.004	0.41	0.18, 0.94	0.034	0.41	0.16, 1.02	0.056
BMI (1 unit increase)	0.86	−1.02, 2.74	0.368									
Malnutrition risk			0.949						0.684			
Moderate cf low risk	1.98	−13.07, 17.04					1.35	0.68, 2.67				
High cf low risk	−1.64	−18.66, 15.38					1.00	0.40, 2.53				
Frailty			0.326						0.942			
Pre-frail cf non-frail	13.87	−4.35, 32.10					0.88	0.40, 1.95				
Frail cf non-frail	3.64	−13.04, 20.31					0.89	0.44, 1.78				
Chronic kidney disease			0.146			0.535			0.045			0.191
Stage 1 cf no CKD	−11.73	−38.40, 14.95		6.16	−26.01, 38.32		4.20	0.57, 30.70		2.80	0.31, 25.00	
Stage 2 cf no CKD	−12.82	−40.79, 15.15		4.68	−21.30, 30.65		3.77	0.42, 33.42		2.36	0.23, 23.95	
Stage 3/4 cf no CKD	−37.12	−71.74, −2.50		−4.89	−40.66, 30.89		15.00	1.58, 142.05		7.88	0.80, 77.71	
Anemia	6.02	−9.74, 21.77	0.452				0.87	0.57, 1.31	0.491			
Loop diuretic	−18.16	−37.72, 1.40	0.069	−16.05	−31.58, −0.52	0.043	1.70	0.90, 3.21	0.101	1.46	0.76, 2.82	0.259
Levothyroxine	25.79	2.84, 48.75	0.028	36.30	−6.84, 79.45	0.099	0.36	0.14, 0.91	0.031	0.32	0.12, 0.90	0.030
ONS	20.31	−10.63, 51.25	0.197	19.69	−33.29, 72.67	0.465	0.55	0.16, 1.86	0.335			

Abbreviations: CI, confidence interval; OR, odds ratio; BMI, Body Mass Index (kg/m^2^); CKD, chronic kidney disease; ONS, oral nutritional supplement. * *n* = 249. ^a^ Variables were included in the adjusted regression models where *p* < 0.25 in the unadjusted models; ^b^ Coefficients from quantile regression with robust clustered (by rest home) standard errors.

**Table 4:** Predictors of serum thyroglobulin concentrations of New Zealand rest home residents *.

	Serum Thyroglobulin Concentration (ng/mL)						Serum Thyroglobulin Concentration >40 ng/mL					
	Unadjusted			Adjusted ^a^			Unadjusted			Adjusted ^a^		
	Coef ^b^	95% CI	*p*-Value	Coef ^b^	95% CI	*p*-Value	OR	95% CI	*p*-Value	OR	95% CI	*p*-Value
Age (5-year increase)	1.46	−0.43, 3.35	0.129	2.59	0.33, 4.86	0.025	1.05	1.01, 1.09	0.021			
Women cf men	4.67	−0.41, 9.75	0.071	6.41	2.36, 10.47	0.002	1.89	1.28, 2.82	0.001			
Date of data collection	−3.43	−8.86, 2.00	0.215	−2.70	−9.65, 4.27	0.447	0.90	0.50, 1.62	0.715			
BMI (continuous)	0.44	0.02, 0.87	0.041	3.43	1.17, 5.70	0.003	1.01	0.97, 1.05	0.600			
Malnutrition risk			0.992						0.252			
Moderate cf low risk	0.78	−11.53, 13.09					0.63	0.30, 1.31				
High cf low risk	0.29	−7.14, 7.72					0.69	0.30, 1.68				
Frailty			0.970						0.029			0.029
Pre-frail cf non-frail	0.33	−6.02, 6.68					3.42	1.23, 9.55		4.08	1.40, 11.96	
Frail cf non-frail	0.67	−4.95, 6.29					1.85	0.75, 4.55		2.46	0.94, 6.45	
Chronic kidney disease			0.057			0.604		0.495				
Stage 1 cf no CKD	6.32	1.02, 11.62		−6.70	−16.54, 3.16		3.80	0.54, 26.56				
Stage 2 cf no CKD	6.38	−1.21, 13.97		−5.95	−16.47, 4.56		4.14	0.46, 37.67				
Stage 3/4 cf no CKD	13.17	−6.05, 32.38		−9.19	−26.93, 8.56		6.11	0.68, 54.56				
Anemia	−5.11	−12.38, 2.16	0.167	−5.85	−11.71, 0.00	0.050	0.39	0.21, 0.73	0.003	0.42	0.20, 0.85	0.016
hs-CRP (log transformed)	1.89	0.29, 3.49	0.021	1.89	−0.06, 3.84	0.057	1.00	0.98, 1.02	0.861			
Loop diuretic	1.58	−2.76, 5.92	0.474				1.15	072, 1.82	0.558			
Levothyroxine	−12.46	−18.60, −6.32	<0.001	−15.22	−24.22, −6.23	0.001	0.53	0.20, 1.40	0.203	0.33	0.09, 1.13	0.078
ONS	−6.60	−12.63, −0.57	0.032	−3.90	−8.89, 1.08	0.124	0.16	0.02, 1.05	0.092	0.42	0.20, 0.85	0.056

Abbreviations: CI, confidence interval; OR, odds ratio; BMI, Body Mass Index (kg/m^2^); CKD, Chronic kidney disease; hs-CRP, high sensitivity c-reactive protein; ONS, oral nutritional supplements. * Participants with elevated thyroid antibodies (*n* = 35) were excluded from analysis; final *n* = 232. ^a^ Variables were included in the adjusted regression models where *p* < 0.25 in the unadjusted models; ^b^ Coefficients from quantile regression with robust clustered (by rest home) standard errors.

**Table 5:** Bread and iodine intake by elderly New Zealand rest home residents before and after iodine fortification of bread *.

Bread Intake (g/Day)				Pre-Fortification Iodine Intake from Bread (μg/Day)		Post-Fortification Iodine Intake from Bread (μg/Day)	
	*n*	Mean	95% CI	Mean	95% CI	Mean	95% CI
All participants	309	66	56, 75	0.7	0.7, 0.8	30.8	26.3, 35.3
Sex							
Men	100	75 ^a^	62, 87	0.7	0.6, 0.9	35.5 ^a^	29.2, 41.8
Women	209	61 ^b^	53, 70	0.7	0.6, 0.8	28.6 ^b^	24.5, 32.6
Age group (years)							
65–80	69	70	54, 86	0.7	0.5, 0.8	33.3	25.4, 41.2
80–89	149	66	59, 74	0.8	0.7, 0.8	31.0	27.3, 34.7
≥90	91	61	49, 73	0.7	0.6, 0.8	28.7	23.0, 34.3
Time of data collection							
Summer/autumn	155	62	56, 67	0.7	0.6, 0.8	28.8	24.3, 33.2
Winter/spring	154	70	64, 76	0.7	0.5, 0.9	32.9	25.8, 40.0
Body Mass Index (kg/m^2^)							
<20	42	51 ^a^	38, 64	0.6	0.4, 0.8	23.8 ^a^	17.5, 30.0
20–24.9	106	65 ^b^	56, 74	0.7	0.7, 0.8	30.5 ^b^	26.3, 34.7
25–29.9	94	64 ^b^	54, 73	0.7	0.6, 0.9	29.7 ^b^	25.3, 34.1
≥30	59	79 ^c^	65, 93	0.8	0.6, 1.0	37.5	30.4, 44.5
Malnutrition risk							
Low risk	205	71 ^a^	62, 80	0.8 ^a^	0.7, 0.9	33.5 ^a^	29.1, 37.9
Moderate risk	53	55 ^b^	43, 66	0.6 ^b^	0.5, 0.7	25.3 ^b^	20.3, 30.4
High risk	37	49 ^b^	36, 62	0.6 ^b^	0.4, 0.8	23.1 ^b^	16.9, 29.3
Frailty							
Not frail	49	75 ^a^	65, 85	0.9 ^a^	0.7, 1.0	35.0 ^a^	27.3, 42.8
Pre-frail	93	68	59, 76	0.7	0.6, 0.8	32.0	27.2, 36.8
Frail	143	61 ^b^	55, 67	0.7 ^b^	0.5, 0.8	28.6 ^b^	24.2, 33.0

* Mandatory fortification of bread with iodized salt was established in New Zealand from September 2009; Postfortification intakes were measured in 2014. ^a,b,c^ Values within a subgroup column with unlike superscript letters were significantly different (*p* < 0.05).
